# High-Performance Automated Detection of Sheep Binocular Eye Temperatures and Their Correlation with Rectal Temperature

**DOI:** 10.3390/ani15172475

**Published:** 2025-08-22

**Authors:** Yadan Zhang, Ying Han, Xiaocong Li, Xueting Zeng, Waleid Mohamed EL-Sayed Shakweer, Gang Liu, Jun Wang

**Affiliations:** 1 Key Laboratory of Smart Agriculture System Integration, Ministry of Education, China Agricultural University, Beijing 100083, China; lyzhzyd@163.com (Y.Z.); hanyingxx@163.com (Y.H.); chatlxc@163.com (X.L.); xueting_cau@163.com (X.Z.); 2 Key Laboratory of Agricultural Information Acquisition Technology, Ministry of Agriculture and Rural Affairs, China Agricultural University, Beijing 100083, China; 3 Animal Production Department, Agricultural and Biological Research Institute, National Research Centre, Cairo 12622, Egypt; shakweer@gmail.com; 4 School of Information Engineering, Henan University of Science and Technology, Luoyang 471003, China

**Keywords:** thermal imaging, bilateral eye temperatures, rectal temperature, statistical analysis

## Abstract

Although rectal temperature is reliable, its measurement requires manual handling and causes stress to animals. IRT provides a non-contact alternative but often ignores bilateral eye temperature differences. This study presents an E-S-YOLO11n model for the automated detection of the binocular regions of sheep, which achieves remarkable performance with a precision of 98.2%, recall of 98.5%, *mAP*@0.5 of 99.40%, *F*_1_ score of 98.35%, FPS of 322.58 frame/s, parameters of 7.27 M, model size of 3.97 MB, and GFLOPs of 1.38. Right and left eye temperatures exhibit a strong correlation (r = 0.8076, *p* < 0.0001), However, the eye temperatures show only very weak correlation with rectal temperature (right eye: r = 0.0852; left eye: r = −0.0359), and neither figure reaches statistical significance. Rectal temperature is 7.37% and 7.69% higher than the right and left eye temperatures, respectively. Additionally, the right eye temperature is slightly higher than the left eye (*p* < 0.01). The study demonstrates the feasibility of combining IRT and deep learning for non-invasive eye temperature monitoring, although environmental factors may limit it as a proxy for rectal temperature. These results support the development of efficient thermal monitoring tools for precision animal husbandry.

## Simple Summary

Rectal temperature measurement is labor-intensive and induces stress in sheep, potentially compromising both measurement accuracy and animal welfare. Infrared thermography (IRT) offers a non-contact alternative with rapid image acquisition and minimal disturbance. However, most existing research focuses on single-point temperature analysis and often overlooks the diagnostic potential of differences between left and right eye temperatures under varying physiological and environmental conditions. This study integrates deep learning with IRT to detect the left and right eye regions of sheep and extract temperature features. An optimized lightweight model (E-S-YOLO11n) was developed, showing high accuracy and fast detection speed on low-power devices. The temperatures of the two eyes are strongly correlated, but neither exhibits a significant correlation with rectal temperature, which is consistently higher. This suggests that eye temperature is influenced by local environmental factors and may not be a reliable surrogate for rectal temperature. These findings offer practical solutions for precision animal husbandry and support more effective scientific herd management.

## 1. Introduction

In various livestock species, eye temperature has been associated with fever, inflammation, heat stress, and emotional responses [1,2]. Among various anatomical sites for surface temperature measurement in livestock, the periocular region is considered an optimal choice due to its unique physiological characteristics, including sparse hair coverage, anatomical exposure, rich blood perfusion, and proximity to the hypothalamic thermoregulatory center [3]. Compared with invasive methods, measuring eye temperature causes less stress and is operationally more convenient, making it suitable for precision livestock farming [4]. Rectal temperature remains the gold standard for core body temperature measurement and is often used as a reference for evaluating the effectiveness of non-contact methods [5]. However, for temperamental individuals or those suffering from rectal diseases, measuring rectal temperature can be difficult, and the procedure may increase the risk of disease transmission [6]. Additionally, measuring rectal temperature often induces stress in sheep, as it requires physical restraint and handling. This stress may adversely impact productivity, immune function, and thermoregulation [7,8]. As a result, non-contact temperature measurement methods, with advantages of speed, non-invasiveness, and reduced stress, are gaining attention as a promising alternative for livestock body temperature monitoring.

IRT is increasingly being adopted in livestock body temperature assessment due to its non-invasive nature, real-time measurement capabilities, and continuous monitoring potential [9]. Compared to other surface regions, eye temperature is more responsive to fluctuations in core body temperature [10]. Current studies often use maximum eye temperature as a key parameter for predicting core body temperature or identifying abnormal physiological states in livestock. Giro et al. [11] developed a linear regression model between maximum right eye temperature and rectal temperature in beef cattle, reporting a relatively low correlation coefficient of 0.392. Chen et al. [12] found that, compared to minimum and average eye temperature or the proportion of scleral white, maximum eye temperature is a more reliable measure of cattle temperament. Additionally, Neto et al. [13] demonstrated the utility of unilateral maximum eye temperature for detecting heat stress in piglets in semi-arid environments. However, unilateral eye temperature is highly susceptible to external environmental factors such as asymmetric lighting, occlusion, wind direction, and head posture, resulting in considerable fluctuations in the measurement results [14,15]. Moreover, physiological asymmetries in brain structure and blood flow distribution can also lead to significant differences between the temperatures of the left and right eyes [16]. These limitations reduce the reliability of single-eye measurements in representing core body temperature or signaling abnormal physiological states. In contrast, a binocular approach allows for cross-validation between both eyes, which can help mitigate errors induced by environmental and physiological variations. By incorporating bilateral temperature data, this method improves the robustness and precision of non-contact temperature assessments in livestock.

Several recent studies have explored the utility of binocular eye temperature for detecting health anomalies in livestock. Wang et al. [17] achieved automated mastitis detection in 50 dairy cows using the temperature difference between the maximum eye and udder temperatures, enabling efficient and non-invasive diagnosis. Uddin et al. [18] reported that the maximum bilateral eye temperatures in 31 dairy cows showed lower variability and greater sensitivity in detecting lesions, infections, and stress, compared to minimum and average values. Similarly, Idris et al. [19] used binocular eye temperature to assess heat stress in 24 Angus steers, showing its potential as a non-invasive heat stress indicator. These studies highlight that differences in binocular eye temperature can reflect physiological or health-related states. However, large-sample analyses are still lacking, making it difficult to generalize the findings to population-level health assessments.

In recent years, the integration of deep learning with IRT has significantly advanced the automation of livestock temperature monitoring. Zhang et al. [20] proposed an improved YOLOv7 model for detecting sheep left eye temperature, reaching high performance with a precision of 99.5%, recall of 99.3%, *mAP*@0.5 of 99.7%, and FPS of 99.3. Wang et al. [21] developed an improved YOLOv8n-mvc model for detecting head temperature in group-housed chickens, achieving a precision of 91.6%, recall of 92.5%, and *F*_1_ of 92.0%, but the FPS was only 23.8 and the parameters were relatively high at 14.44M. Zhang et al. [22] proposed a lightweight YOLOv7-tiny-Ghost model for pig eye temperature detection, attaining a precision of 95.1%, recall of 95.4%, and *mAP*@0.5 of 95.4%, while maintaining a small parameter (3.59 M) and high inference speed (119.05 FPS). The synergy between deep learning and IRT not only improves operational efficiency by accelerating data acquisition and enhancing detection accuracy but also minimizes physiological stress in livestock. This combination is emerging as a transformative approach in modern animal health surveillance. Simultaneously, to facilitate practical deployment in agricultural edge computing environments, contemporary models are undergoing systematic lightweight optimization through architectural innovations. Nonetheless, existing research predominantly focuses on single-target temperature analysis and often neglects the diagnostic potential of bilateral eye temperature differences under dynamic physiological and environmental conditions.

Investigating the correlation and differences between surface and rectal temperatures is essential for evaluating the accuracy and reliability of non-contact temperature measurements in livestock [23]. Stumpf et al. [24] established a regression model between udder surface temperature and rectal temperature and found that the maximum udder temperature exhibited the highest coefficient of determination (*p* < 0.01), indicating a certain predictive potential. Easterwood et al. [25] employed Bland–Altman analysis to assess the agreement between forehead and neck surface temperatures and rectal temperature in horses, revealing a significant systematic bias. Hoffman et al. [26] explored the feasibility of replacing rectal temperature with ocular temperature in steers and reported Pearson and Spearman correlation coefficients of 0.71 and 0.66, respectively (*p* < 0.01). However, existing studies on the relationship between surface and rectal temperatures in livestock often lack a comprehensive analysis of data distribution, and thus, there remains a need for further validation of the observed correlations and differences. Moreover, relying solely on a single statistical analysis or regression model is insufficient to robustly support IRT as a viable alternative for livestock body temperature monitoring.

This study aims to improve the accuracy and efficiency of automated sheep eye temperature detection, evaluate the correlation between eye and rectal temperatures, and analyze the differences between bilateral eye temperature and rectal temperature. This study makes the following specific contributions:

1. The E-S-YOLO11n model was proposed for automated detection of the binocular eye region in sheep;
2. Statistical methods were chosen based on tests of normality and variance homogeneity for eye temperature and rectal temperature;
3. Pearson correlation analysis was performed to evaluate the relationship between bilateral eye temperature and rectal temperature;
4. An independent samples t-test was conducted to examine the differences between bilateral eye temperature and rectal temperature.

## 2. Materials and Methods

### 2.1. Data Collection

**Figure 1:**
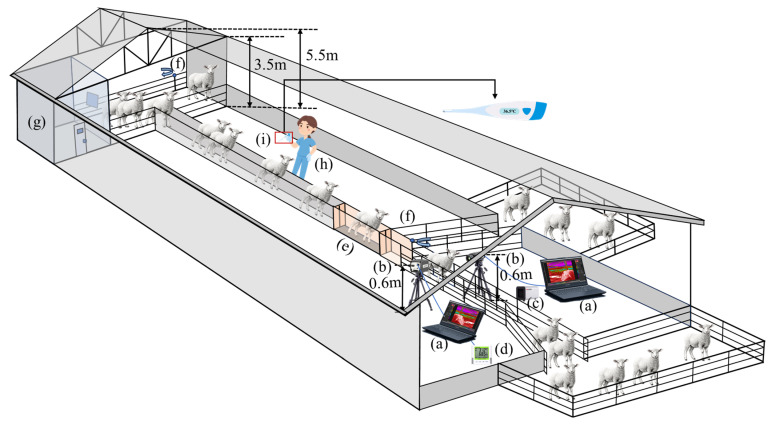
The data collection system. (**a**) Computer; (**b**) thermal imager; (**c**) blackbody; (**d**) hygrothermograph; (**e**) weight scale; (**f**) revolving door; (**g**) veterinary operating room; (**h**) measurement of rectal temperature; (**i**) veterinary thermometer.

From 24 September to 4 October 2024, a data collection experiment on sheep binocular infrared video and rectal temperature measurement was conducted at Mengzhiyuan Livestock Company in Xilinhot City, Inner Mongolia Autonomous Region, China. The experimental sheep were crossbred offspring of Ujumqin ewes and Dorper rams. A total of 1600 clinically healthy female sheep were selected. Their ages ranged from 1.5 to 2 years, and their body weights ranged from 45 to 55 kg. Prior to the experiment, all sheep underwent veterinary examination to ensure the absence of any health conditions that could affect body temperature measurements, such as fever, respiratory abnormalities, or ocular lesions. During the experiment, all sheep were subjected to a consistent husbandry and management protocol. Each day, the sheep were herded from a semi-open barn to a pasture at 5:00 a.m. for free grazing and returned to the barns at 5:00 p.m. Clean drinking water was available during both grazing and housing periods. The uniformity of the sheep flock in age, sex, health status, and husbandry conditions helped minimize potential confounding factors affecting rectal and eye temperature measurements, thereby enhancing the reliability of subsequent temperature correlation analyses. The data collection system is shown in Figure 1. During the experiment, rectal temperature was measured using a veterinary thermometer (Model MAT-1, Shangrao Maodeng Science and Technology Limited Company, Shangrao, China, measurement range: 32–45 °C, accuracy: ±0.1 °C, operating temperature: −10–40 °C) while the sheep were in the handling passage. The thermometer emitted an audible signal once the temperature reading had stabilized for at least 15 s. After passing through the handling passage, each sheep’s binocular infrared thermal video was sequentially captured using two FLIR A310 thermal imagers (FLIR Systems, Inc., Wilsonville, OR, USA). The imagers were configured with an emissivity of 0.98 and a frame rate of 15. Both A310 thermal imagers were mounted on tripods at a height of 0.6 m and positioned 0.8 m away from the passage. A MIEYE-50 blackbody was set to 37 °C to ensure accurate temperature calibration. Additionally, a HOBO hygrothermograph (Onset Computer Corporation, Bourne, MA, USA) was connected to a computer, utilizing HOBOware software (Version 3.7.18) to continuously record environmental temperature and humidity in real time at 5 min intervals.

During the experiment, a total of 2248.5 min of sheep binocular infrared thermal video was recorded. The 773 min of infrared thermal videos from non-estrus sheep were frame-extracted, resulting in 17,170 thermal images of sheep with a resolution of 320 × 240. After screening these images, thermal images of 1165 sheep with a fully visible binocular region were selected. Among them, 106 sheep had both binocular thermal images and corresponding rectal temperatures. The dataset was divided into training, validation, and test sets in a 7:2:1 ratio based on individual sheep, as shown in Table 1. This resulted in 848 sheep (1696 thermal images) in the training set, 211 sheep (422 thermal images) in the validation set, and 106 sheep (212 thermal images) in the test set.

### 2.2. Improved YOLO11 Network Framework

**Figure 2:**
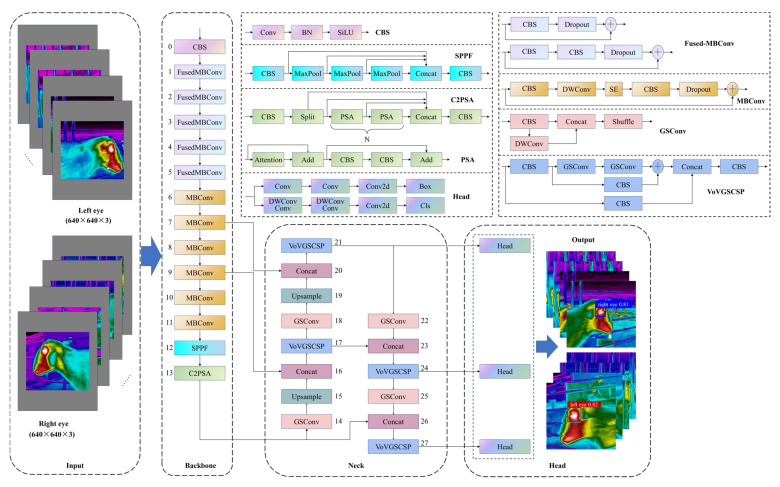
E-S-YOLO11n network architecture.

As a high-precision and high-efficiency object detection model, YOLO11 offers significant improvements in detection performance, inference speed, and generalization ability compared to other models in the YOLO series. Additionally, it exhibits superior adaptability to diverse environments and better compatibility with edge devices. To accurately detect the eyes of sheep and achieve high-precision classification, this study designs a Backbone network based on EfficientNetV2 to enhance the extraction of thermal infrared features from sheep eyes [27]. Furthermore, a Slim-Neck structure is introduced into the Neck network, effectively improving detection accuracy while reducing computational complexity [28]. The improved YOLO11 model (E-S-YOLO11n), incorporating EfficientNetV2 and Slim-Neck, consists of three main components: Backbone, Neck, and Head. The overall network architecture is illustrated in Figure 2.

### 2.3. Backbone Network

The grayscale-padded thermal images of sheep eyes, with a resolution of 640 × 640, are fed into the Backbone network. The Backbone, based on EfficientNetV2, is designed to extract thermal infrared features of sheep eyes. It consists of CBS, Fused-MBConv, MBConv, SPPF, and C2PSA blocks. Through the Backbone network, the thermal images undergo feature extraction at three effective feature layers: the 7th layer (40 × 40 × 128), the 9th layer (20 × 20 × 160), and the 13th layer (10 × 10 × 1024), ensuring effective feature representation.

The CBS block consists of 2D convolution (Conv2D), batch normalization (BN), and the SiLU activation function. The Fused-MBConv block comprises a CBS with a 3 × 3 convolution, a CBS with a 1 × 1 convolution, and a stochastic depth module. The 3 × 3 convolutional CBS expands the number of channels, while the 1 × 1 convolutional CBS compresses them. The stochastic depth block (Dropout) reduces network depth, and its output is summed with the residual connection. Notably, the residual connection and Dropout are only present when the stride is 1 and the input and output channel numbers are equal. Additionally, when the expansion ratio is set to 1, the 3 × 3 convolutional CBS is omitted. In the first layer of the Backbone network, the expansion ratio is 1 with identical input and output channel numbers. In layers 2 to 5, the expansion ratio increases to 4, expanding the number of channels to four times that of the input. For layers 3 and 5, where the stride is 1 and the input and output channels are equal, residual connections are retained to enhance feature reuse.

The MBConv block consists of a CBS with a 1 × 1 convolution, a depthwise convolution (DWConv) with a 3 × 3 kernel (including BN and SiLU), a squeeze-and-excitation (SE) module, a standard 1 × 1 convolution (including BN), and a Dropout. The 1 × 1 CBS expands the number of channels, while the 3 × 3 DWConv extracts high-dimensional spatial features of the sheep’s eyes. The SE module integrates these high-dimensional features along the channel dimension, enhancing inter-channel expression. The standard 1 × 1 convolution compresses the number of channels after fusion. The conditions for the presence of the residual connection and Dropout are the same as in the Fused-MBConv module. In the Backbone network, layers 6 to 11 incorporate the SE module with a compression ratio of 0.25. Layers 6, 7, 10, and 11 have an expansion ratio of 4, while layers 8 and 9 have an expansion ratio of 6, expanding the number of channels to four and six times that of the input, respectively. Additionally, in layers 7, 9, and 11, where the stride is 1 and the input and output channel numbers are identical, both the residual connection and Dropout are present.

### 2.4. Neck Network

The Neck network consists of GSConv, VoVGSCSP, Upsample, and Concat modules. The three effective feature layers extracted by the Backbone network (layers 7, 9, and 13) are processed through the Neck network, generating three multi-scale fused thermal infrared feature layers for sheep eyes: layer 21 (40 × 40 × 256), layer 24 (20 × 20 × 512), and layer 27 (10 × 10 × 1024). This enhances the extraction and representation of thermal infrared features in sheep eyes.

GSConv integrates a DWConv block and a Shuffle layer to enhance the nonlinear expressiveness of the lightweight detection model. The input thermal infrared feature map of the sheep eye first passes through the CBS and DWConv modules, where the CBS module extracts dense channel features, while the DWConv module extracts sparse channel features. The dense and sparse channel features are then concatenated, and the Shuffle layer facilitates uniform feature fusion across channels, optimizing channel feature representation. GSConv strengthens inter-channel connections, improving feature expression while reducing computational cost. However, applying GSConv across all stages of the YOLO11 model would result in a denser network structure, increasing the difficulty of extracting thermal infrared features from sheep eyes and prolonging model inference time. As feature maps progress through the Neck network, their channel dimensions gradually increase while their spatial dimensions (width and height) decrease. Therefore, GSConv is specifically employed in the Neck network to prevent redundancy in feature fusion and maintain efficient information integration.

The efficient cross-stage partial block (VoVGSCSP) in the Neck network facilitates efficient fusion of thermal infrared features for sheep eyes. The input thermal infrared feature map is divided into two branches within VoVGSCSP. The first branch employs a 1 × 1 convolutional CBS module to reduce the number of output channels to half of the input channels. The compressed feature map is then fed into the GSbottleneck block for deep feature extraction of sheep eyes. Meanwhile, the second branch utilizes another 1 × 1 convolutional CBS module to extract shallow features. The deep and shallow features are concatenated along channels. Then, a 1 × 1 convolutional CBS is used to integrate the channel information of the concatenated sheep eye features, reducing the channel dimensionality and generating a more expressive feature map for sheep eyes.

### 2.5. Head Network

The Head network consists of two primary branches, including bounding box regression prediction and category probability prediction. The bounding box regression branch extracts bounding box features using two 3 × 3 convolutions and then predicts the discrete distribution of bounding box parameters using a 1 × 1 convolution. The total number of channels of the bounding box regression branch is 4 × *reg*_max. To enhance prediction accuracy, distribution focal loss is applied to convert the discrete bounding box parameters into continuous values. The category probability prediction branch consists of two sub-blocks and a 1 × 1 convolution. Each sub-block comprises a 3 × 3 DWConv followed by a 1 × 1 standard convolution, responsible for extracting classification features for sheep eyes. The final 1 × 1 convolution outputs two channels, representing the probability of the detected feature belonging to either the left or right eye.

For each anchor, the total output dimension no is calculated as follows:

(1) no=nc+4×reg_max

where the category channel (*nc*) is 2, and the maximum regression range (*reg*_max) is set to 16.

### 2.6. Experimental Platform

The hardware platform used for this experiment is configured with an Intel (R) Core (TM) i9-14900KF 3.20 GHz processor, 32.0 GB of operating memory, an NVIDIA GeForce RTX 4080 SUPER GPU, the CUDA 12.3 parallel computing framework, the Python v.3.10 programming language, and the Pytorch 2.3.0 deep learning framework. The model training hyperparameters are set as follows: the training process utilizes an automatically selected optimizer, with 300 training epochs, an initial learning rate of 0.01, a learning rate factor of 0.01 (resulting in a final learning rate of 0.0001), a batch size of 16, and an optimizer weight decay of 0.0005.

### 2.7. Evaluation Indicators

To evaluate the performance of the E-S-YOLO11n model in the thermal image dataset of sheep eyes, the model’s predictive results are assessed using precision (*P*), recall (*R*), *F*_1_ score (*F*_1_), and mean average precision (*mAP*). The intersection over union threshold for model detection is set to ≥0.5. The evaluation indicators are defined as follows:

(2) $$P=\frac{TP}{TP+FP}\times 100\%$$

(3) $$R=\frac{TP}{TP+FN}\times 100\%$$

(4) $$F_{1}=\frac{2TP}{2TP+FP+FN}\times 100\%$$

(5) $$mAP=\frac{1}{N}\sum_{i=1}^{N}AP_{i}\times 100\%$$

where *TP* represents the number of correctly detected sheep eyes by the model, *FN* denotes the number of missed detections, and *FP* indicates the number of falsely detected sheep eyes. *N* refers to the number of categories, while *AP* is the area under the interpolated *P*-*R* curve enveloped with the X-axis.

### 2.8. Algorithm Comparison

On the same dataset (as shown in Table 1), the precision, recall, *F*_1_, *mAP*@0.5, FPS, parameters, model size, and GFLOPs of the YOLO11 series models were obtained to compare model performance and select an appropriate baseline model. To evaluate the performance of the E-S-YOLO11n model in detecting sheep eyes in thermal images, its precision, recall, *F*_1_, *mAP*@0.5, FPS, parameters, model size, and GFLOPs were compared against YOLO11n, YOLO10n, YOLOx, Centernet, SSD, and Faster R-CNN using the same dataset. The manually annotated and automatically detected sheep eye categories (right eye: category ID 0, left eye: category ID 1) and bounding box coordinates (center x, y, width w, height h) were input into the thermal image temperature extraction program Sheep temperature ext.exe to obtain the maximum eye temperature of each sheep. The extracted maximum eye temperature of each sheep was calibrated using the blackbody temperature and ambient temperature and humidity. Boxplots were generated using both manually annotated and model-detected eye temperatures to analyze the distribution and dispersion of sheep eye temperatures while identifying and filtering outliers.

### 2.9. Statistical Analysis

After filtering the sheep eye temperatures, rectal temperatures were matched with both manually annotated and E-S-YOLO11n model-detected binocular eye temperatures. The differences between rectal temperature and eye temperature were then calculated. Normality and homogeneity of variance tests were conducted on the following temperature variables: manually annotated right eye temperature (True-Right), model-detected right eye temperature (E-S-YOLO11n-Right), manually annotated left eye temperature (True-Left), model-detected left eye temperature (E-S-YOLO11n-Left), rectal temperature minus manually annotated right eye temperature (True-Right-diff), rectal temperature minus model-detected right eye temperature (E-S-YOLO11n-Right-diff), rectal temperature minus manually annotated left eye temperature (True-Left-diff), rectal temperature minus model-detected left eye temperature (E-S-YOLO11n-Left-diff), and rectal temperature (RT). To assess the normality of sheep eye temperature, rectal temperature, and temperature differences, the Kolmogorov–Smirnov (K-S) test was applied, along with visual inspections using histograms and Q-Q plots. Since the K-S test is sensitive to large sample sizes and may detect minor deviations from normality as statistically significant, visual methods were included to provide a more balanced assessment. Homogeneity of variance was evaluated using Levene’s test. Based on the temperature distributions, appropriate correlation analysis and the difference test were selected to examine the relationships and differences between binocular eye temperatures, rectal temperature, and their temperature differences. The correlation coefficient (r) quantifies the strength of association between variables, ranging from −1 to 1. A correlation of |r| = 1 indicates a perfect correlation, 0.8 ≤ |r| < 1 represents a strong correlation, 0.5 ≤ |r| < 0.8 indicates a moderate correlation, 0.3 ≤ |r| < 0.5 signifies a low correlation, and 0 ≤ |r| < 0.3 suggests a weak or negligible correlation.

## 3. Results

Table 2 presents the performance of the YOLO11 model in detecting sheep eyes in thermal images. YOLO11n demonstrated the highest precision (95.6%), *F*_1_ (96.05%), and FPS (312.50 frame/s), outperforming YOLO11s, YOLO11m, YOLO11l, and YOLO11x. Specifically, YOLO11n’s precision surpassed these models by 0.31%, 2.69%, 15.88%, and 2.14%, respectively, while its *F*_1_ exceeded them by 0.11%, 3.56%, 11.87%, and 1.28%. In terms of computational efficiency, YOLO11n exhibited a significantly higher processing speed, with YOLO11s, YOLO11m, YOLO11l, and YOLO11x achieving 51.52%, 52.24%, 61.90%, and 67.01% lower FPS, respectively. Additionally, YOLO11n featured the smallest parameters (9.85M), smallest model size (5.23MB), and lowest GFLOPs (3.20) among all variants. Compared to YOLO11s, YOLO11m, YOLO11l, and YOLO11x, YOLO11n reduced its parameters by 72.57%, 87.11%, 89.78%, and 95.46%, respectively, while its model size was 71.42%, 86.45%, 89.28%, and 95.20% smaller. Similarly, its GFLOPs were reduced by 70.19%, 90.57%, 92.64%, and 96.72%, respectively. Notably, YOLO11n achieved a recall of 96.5%, only 0.10% lower than YOLO11s. Its *mAP*@0.5 reached 97.8%, slightly below YOLO11s and YOLO11x by 1.11% and 0.81%, respectively. Despite these minor trade-offs, YOLO11n was chosen as the baseline model due to its exceptional balance of high precision, recall, *mAP*@0.5, and *F*_1_ (all above 95%), while maintaining the highest FPS and the lowest parameters, model size, and GFLOPs. Although YOLO11n shows slightly lower *mAP*@0.5 and recall compared to some larger models, it still maintains excellent precision and robustness under dynamic field conditions. More importantly, its superior inference speed and computational efficiency make it the most practical choice. Therefore, YOLO11n was chosen as the baseline model based on a comprehensive trade-off between performance, robustness, and deployment feasibility.

The performance of E-S-YOLO11n, YOLO11n, YOLO10n, YOLOx, Centernet, SSD, and Faster R-CNN in sheep eye detection was evaluated using precision, recall, *F*_1_, *mAP*@0.5, FPS, parameters, model size, and GFLOPs, as shown in Table 3. Among all models, E-S-YOLO11n achieved the highest precision, recall, *mAP*@0.5, and *F*_1_ while maintaining the lowest parameters, smallest model size, and lowest GFLOPs. However, its FPS was 25.81% lower than that of YOLO10n. Compared to YOLO11n, E-S-YOLO11n improved precision, recall, *mAP*@0.5, *F*_1_, and FPS by 2.72%, 2.07%, 1.64%, 2.40%, and 3.23%, respectively, while reducing the parameters, model size, and GFLOPs by 26.18%, 24.09%, and 57.03%, respectively. Among the tested models, SSD exhibited the lowest precision (69.91%), which was 28.81% lower than that of E-S-YOLO11n. YOLOx had the lowest recall (58.49%), *mAP*@0.5 (77.66%), and *F*_1_ (64.34%), which were 40.62%, 21.87%, and 34.58% lower than those of E-S-YOLO11n, respectively. Centernet had the highest parameters (124.60M) and the largest model size (124.00MB), whereas E-S-YOLO11n reduced these by 94.16% and 96.80%, respectively. Faster R-CNN demonstrated the lowest FPS (37.64 frames/s), which was 88.33% lower than that of E-S-YOLO11n, and the highest GFLOPs, with E-S-YOLO11n achieving a 99.71% reduction in GFLOPs. Overall, compared to YOLO11n, E-S-YOLO11n exhibited superior performance in sheep eye detection, achieving higher precision, recall, *mAP*@0.5, *F*_1_, and FPS while significantly reducing the parameters, model size, and GFLOPs, validating the effectiveness of its lightweight optimization.

**Figure 3:**
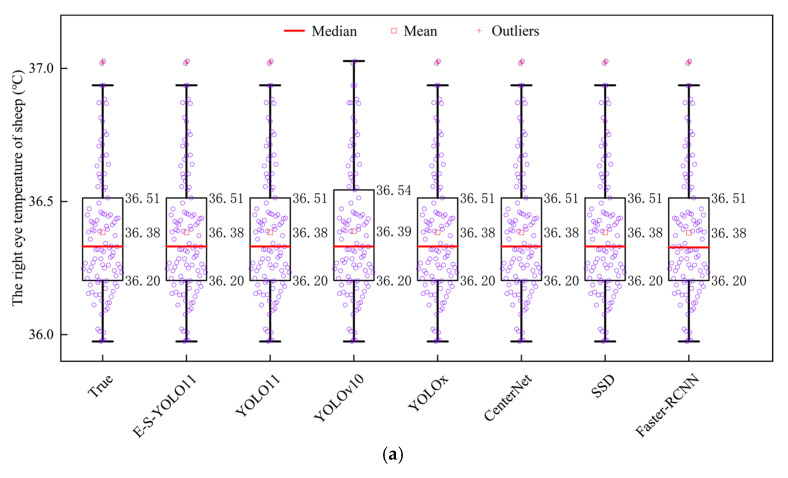

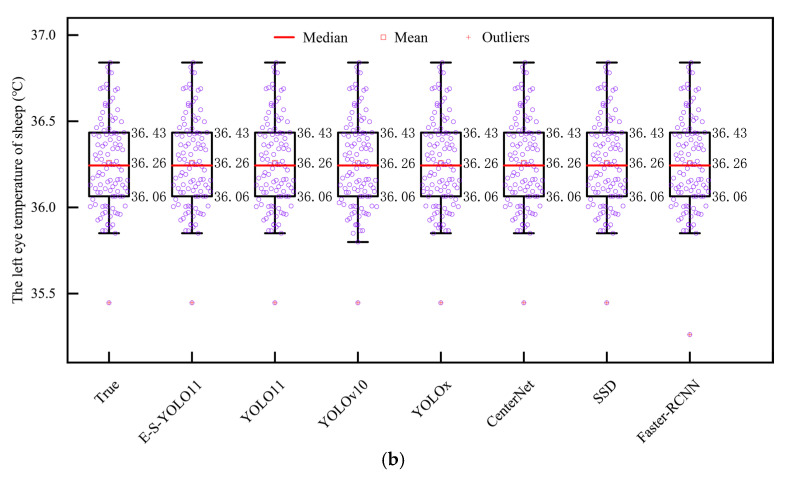
Boxplots of sheep eye temperatures. (**a**) Boxplot of the right eye temperature in sheep; (**b**) boxplot of the left eye temperature in sheep.

Figure 3 presents the boxplots of sheep eye temperatures based on manual annotation and model detection. In Figure 3a, which depicts the boxplot of right eye temperature, the temperature range detected by manual annotation and the E-S-YOLO11n, YOLO11n, YOLO10n, YOLOx, Centernet, SSD, and Faster R-CNN was consistently between 35.97 °C and 37.03 °C. The right eye temperature detected by manual annotation, E-S-YOLO11n, YOLO11n, YOLOx, Centernet, SSD, and Faster R-CNN was 36.38 ± 0.24 °C, while YOLO10n reported a slightly higher value of 36.39 ± 0.25 °C. The boxplot length indicates that most right eye temperatures were concentrated within the range of 36.20 °C to 36.51 °C. Outliers in right eye temperature were located on the upper limit, specifically at 37.03 °C and 37.02 °C. The median is significantly lower than the center of the interquartile range, indicating that the distribution of manually annotated and model-detected right eye temperatures in sheep exhibits positive skewness. In Figure 3b, which illustrates the boxplot of left eye temperature, the temperature range detected by manual annotation, E-S-YOLO11n, YOLO11n, YOLO10n, YOLOx, Centernet, and SSD was between 35.45 °C and 36.84 °C, whereas Faster R-CNN detected a slightly wider range of 35.26 °C to 36.84 °C. The left eye temperature detected by manual annotation, E-S-YOLO11n, YOLO11n, Centernet, and SSD was 36.26 ± 0.26 °C, while YOLO10n, YOLOx, and Faster R-CNN reported 36.26 ± 0.27 °C. The boxplot length suggests that most left eye temperatures were concentrated between 36.06 °C and 36.43 °C. Outliers in left eye temperature were positioned at the lower limit, measuring 35.45 °C and 35.26 °C, respectively. The median was slightly skewed toward the lower quartile rather than centered, indicating a positively skewed distribution in both manually annotated and model-detected left eye temperatures. Additionally, the maximum temperature reflects the thermal intensity of only a single pixel in the thermal image, making it insensitive to localization errors in the eye region. Consequently, the extracted maximum temperature values fail to effectively highlight performance differences between the models.

After removing outliers from the sheep binocular temperatures, a final dataset of 103 sheep was obtained. Following this outliers cleaning process, the right eye temperature detected by manual annotation and the E-S-YOLO11n, YOLO11n, YOLOx, Centernet, SSD, and Faster R-CNN models was consistently 36.37 ± 0.23 °C, while the YOLO10n model reported a slightly higher right eye temperature of 36.38 ± 0.23 °C. The left eye temperature detected by manual annotation, E-S-YOLO11n, YOLO11n, YOLO10n, YOLOx, Centernet, SSD, and Faster R-CNN was consistently 36.26 ± 0.25 °C.

**Figure 4:**
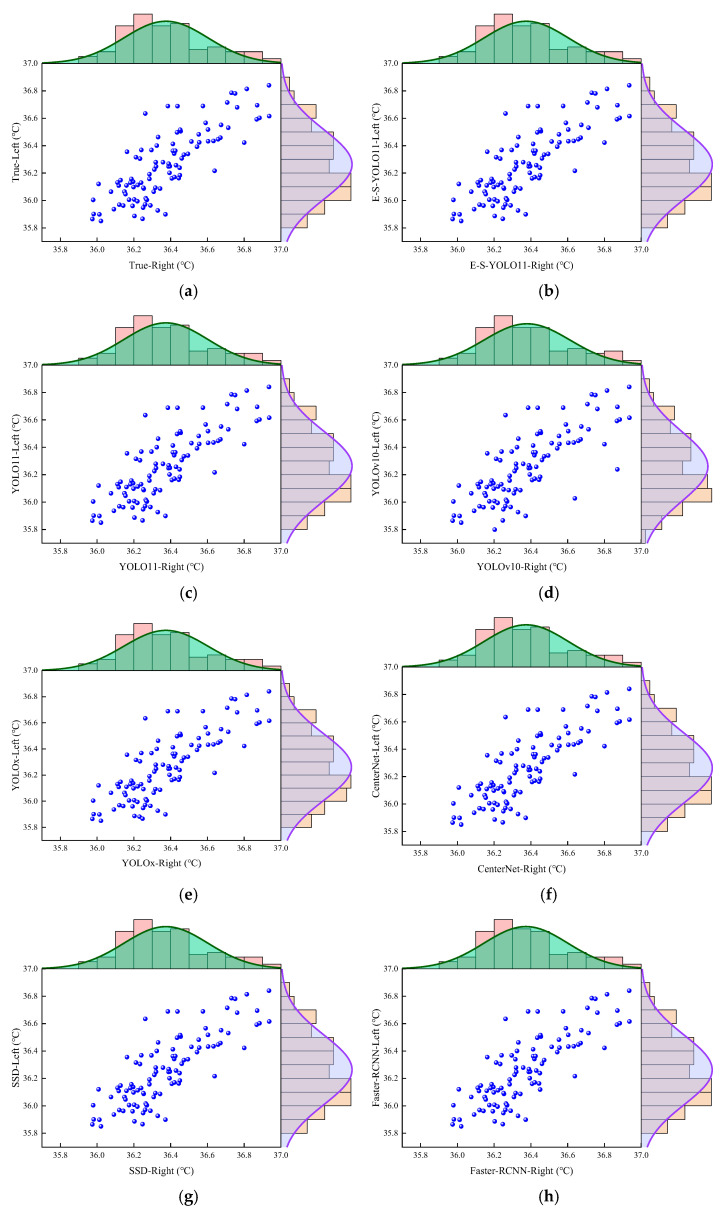
Marginal histograms of sheep eye temperatures. (**a**) Manually annotated sheep eye temperatures; (**b**) sheep eye temperatures detected by E-S-YOLO11n; (**c**) sheep eye temperatures detected by YOLO11n; (**d**) sheep eye temperatures detected by YOLO10n; (**e**) sheep eye temperatures detected by YOLOx; (**f**) sheep eye temperatures detected by CenterNet; (**g**) sheep eye temperatures detected by SSD; (**h**) sheep eye temperatures detected by Faster R-CNN.

Figure 4 presents the marginal histograms of the sheep eye temperatures. The scatter plots of manually annotated and model-detected eye temperatures show that the data are generally aligned along the diagonal, indicating no significant systematic bias between manual annotation and model detection for eye temperatures. A strong positive correlation is observed between left and right eye temperatures, suggesting that the relationship between the two aligns with physiological expectations. The kernel density curve of right eye temperature reveals a unimodal, slightly skewed distribution, with a peak near 36.3 °C. Similarly, the left eye temperature follows a unimodal distribution with a concentrated peak around 36.2 °C, showing a slightly lower temperature than the right eye. Notably, in the scatter plot of YOLO10n-detected temperatures (Figure 4d), data are more widely dispersed, with some deviating significantly from the main trend line, indicating a higher detection error for the YOLO10n model in sheep eye detection. Additionally, the kernel density curve for left eye temperature detected by YOLO10n is wider than those of other models, suggesting greater temperature variation and lower detection precision.

**Figure 5:**
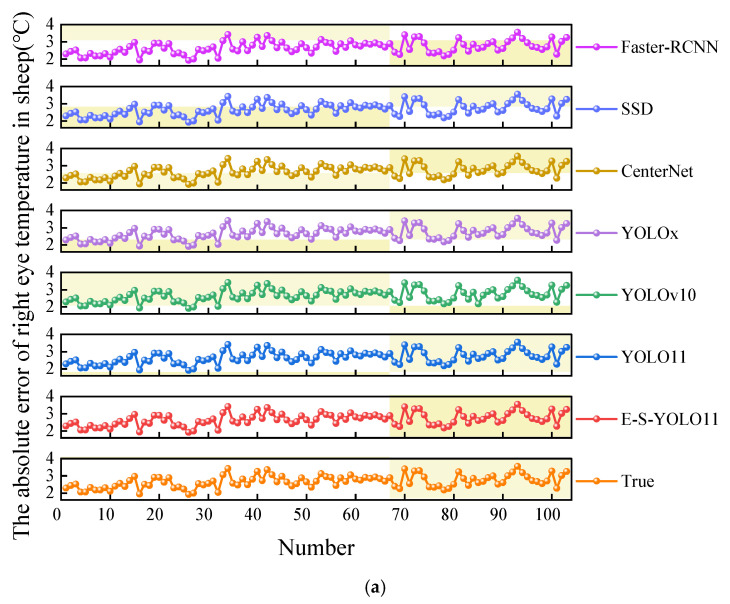

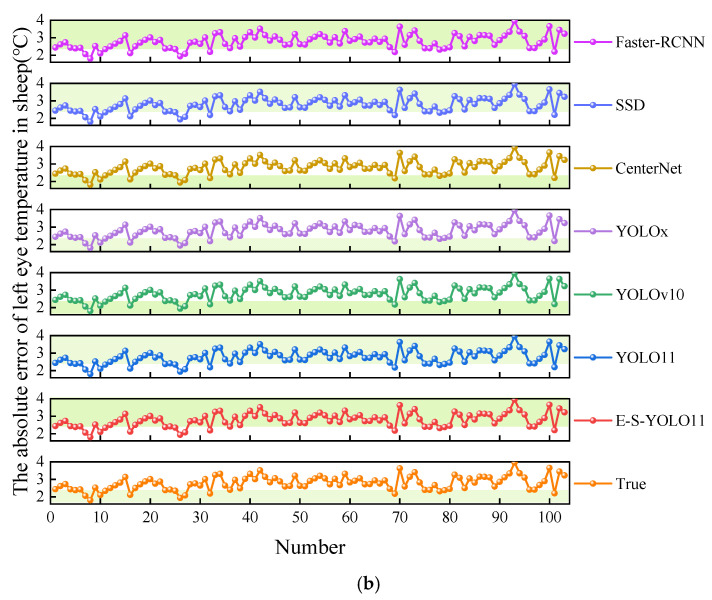
Absolute error of sheep eye temperature and rectal temperature. (**a**) Absolute error between right eye temperature and rectal temperature; (**b**) Absolute error between left eye temperature and rectal temperature.

The absolute error between sheep eye temperature and rectal temperature is illustrated in Figure 5. The mean absolute error (MAE) for right eye temperature detected by manual annotation, E-S-YOLO11n, YOLO11n, YOLOx, CenterNet, and SSD was 2.67 ± 0.36 °C. In contrast, the MAE for right eye temperature detected by the YOLO10n and Faster R-CNN models was 2.66 ± 0.37 °C and 2.68 ± 0.36 °C, respectively. Notably, YOLO10n achieved the lowest MAE (2.66 °C) among all models. However, it mistakenly identified the blackbody in thermal images as the sheep’s right eye, resulting in misclassified right eye temperatures. These misclassifications had a smaller absolute difference from rectal temperature, artificially lowering the MAE. For left eye temperature, the MAE detected by manual annotation, E-S-YOLO11n, YOLO11n, CenterNet, and SSD models was 2.78 ± 0.39 °C. Meanwhile, the MAE for YOLO10n, YOLOx, and Faster R-CNN was 2.79 ± 0.41 °C, 2.79 ± 0.40 °C, and 2.78 ± 0.40 °C, respectively. Furthermore, YOLO10n exhibited the highest standard deviation in absolute error, with 0.37 °C for right eye temperature and 0.41 °C for left eye temperature, surpassing those of manual annotation, E-S-YOLO11n, YOLO11n, YOLOx, CenterNet, SSD, and Faster R-CNN. This indicates that YOLO10n’s absolute error in sheep eye temperature was more dispersed, highlighting its lower detection stability compared to manual annotation and the other models. The clinically acceptable error margin for livestock temperature measurement via IRT is typically ±1.0 °C in practical applications. However, in dynamic, non-contact settings, an MAE of 2.66 °C to 2.79 °C remains within an acceptable practical threshold. Future work will focus on improving detection precision through temperature calibration and physiological modeling.

**Figure A1:**
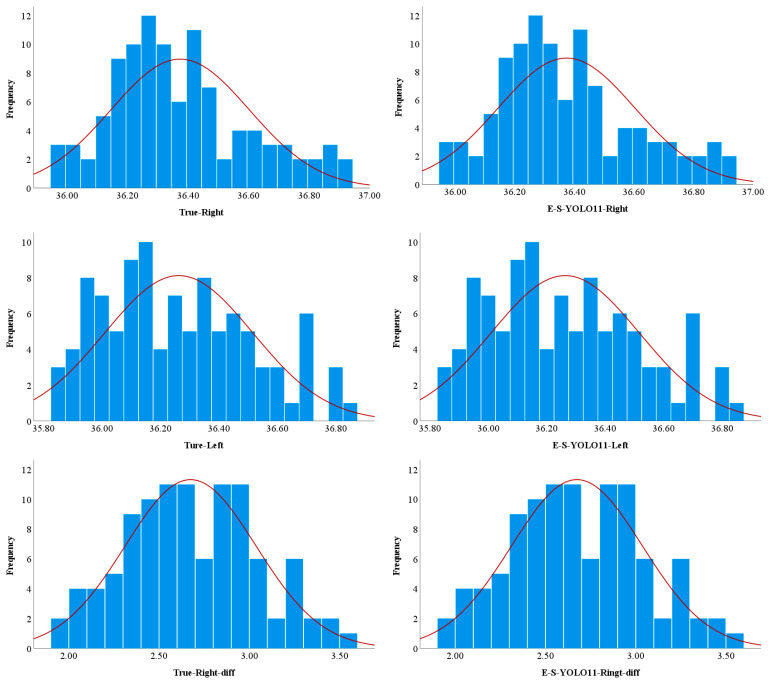

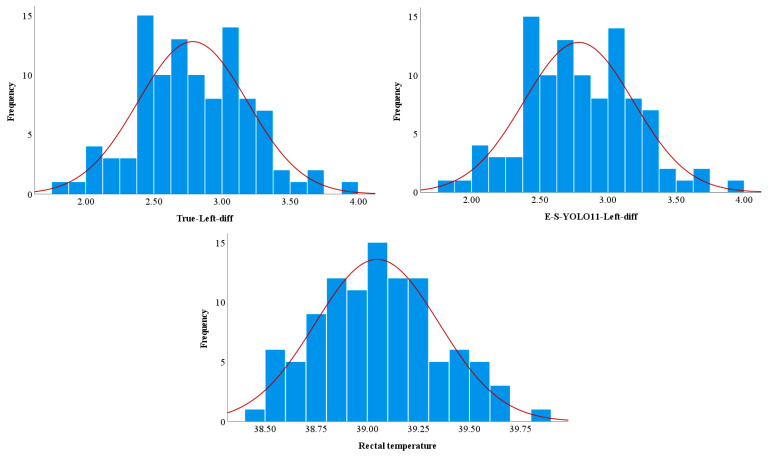
The histograms for sheep eye temperature, rectal temperature, and their differences.

**Figure A2:**
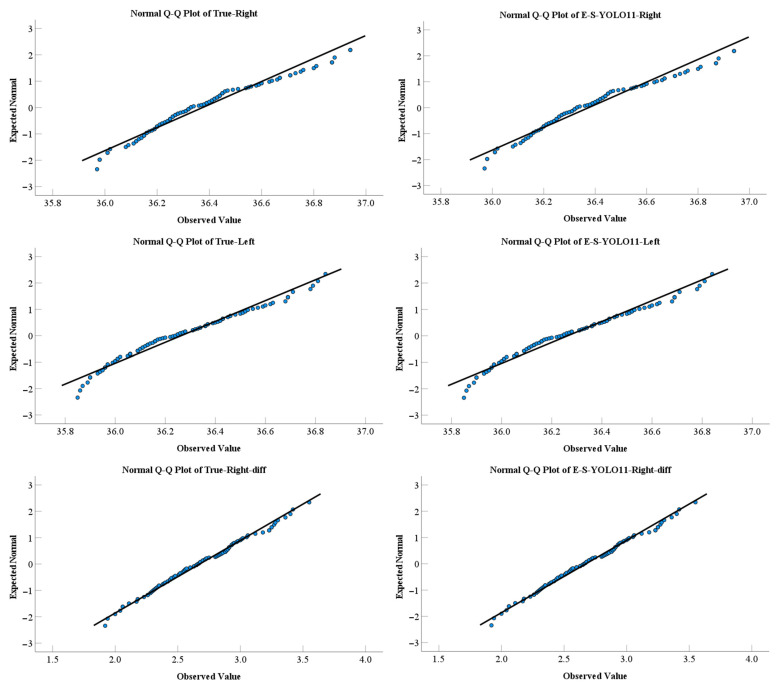

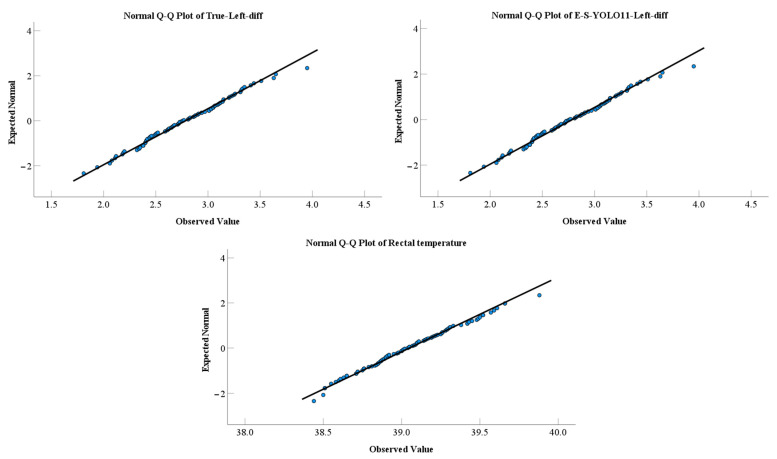
The Q-Q plots for sheep eye temperature, rectal temperature, and their differences.

Table 4 presents the results of the Kolmogorov–Smirnov (K-S) test for sheep eye temperature, rectal temperature, and their differences. The test indicates that True-Right (*p* = 0.017), E-S-YOLO11n-Right (*p* = 0.017), True-Left (*p* = 0.023), and E-S-YOLO11n-Left (*p* = 0.023) all have *p*-values below 0.05, suggesting that these variables do not follow a normal distribution. In contrast, True-Right-diff (*p* = 0.200), E-S-YOLO11n-Right-diff (*p* = 0.200), True-Left-diff (*p* = 0.200), E-S-YOLO11n-Left-diff (*p* = 0.200), and RT (*p* = 0.200) all have P-values greater than 0.05, indicating that they conform to a normal distribution. As shown in Figure A1, the histograms further support these findings. True-Right, E-S-YOLO11n-Right, True-Left, and E-S-YOLO11n-Left exhibit slight right-skewness, while True-Right-diff, E-S-YOLO11n-Right-diff, True-Left-diff, E-S-YOLO11n-Left-diff, and RT appear closer to a normal distribution. Additionally, the Q-Q plots in Figure A2 provide further validation. Data points for True-Right, E-S-YOLO11n-Right, True-Left, and E-S-YOLO11n-Left align closely along the diagonal but show minor deviations at the tails, indicating slight departures from normality. In contrast, the data points for True-Right-diff, E-S-YOLO11n-Right-diff, True-Left-diff, E-S-YOLO11n-Left-diff, and RT follow the diagonal more consistently, reinforcing their normal distribution characteristics. In summary, True-Right, E-S-YOLO11n-Right, True-Left, and E-S-YOLO11n-Left exhibit slight right-skewness, whereas True-Right-diff, E-S-YOLO11n-Right-diff, True-Left-diff, E-S-YOLO11n-Left-diff, and RT follow a normal distribution.

Table 5 presents Levene’s test for homogeneity of variance for sheep eye temperature, rectal temperature, and their differences. The test results show that True-Right (*p* = 0.863), E-S-YOLO11n-Right (*p* = 0.863), True-Left (*p* = 0.055), E-S-YOLO11n-Left (*p* = 0.055), True-Right-diff (*p* = 0.503), E-S-YOLO11n-Right-diff (*p* = 0.503), True-Left-diff (*p* = 0.581), E-S-YOLO11n-Left-diff (*p* = 0.581), and RT (*p* = 0.356) all have *p*-values greater than 0.05, indicating that the variance across groups is homogeneous. These results confirm that both manually annotated and E-S-YOLO11n-detected sheep eye temperatures, rectal temperatures, and their differences all satisfy the assumption of homogeneity of variance.

Based on the normality and homogeneity of variance tests for sheep eye temperature, rectal temperature, and their differences, parametric methods were chosen for further analysis. For correlation analysis of eye temperature, rectal temperature, and temperature differences, the Pearson correlation coefficient was employed, as it is appropriate for normally distributed data and effectively measures the linear relationship between variables. For comparative analysis, the independent samples t-test was used to determine whether there were significant differences between the two independent sample groups.

**Figure 6:**
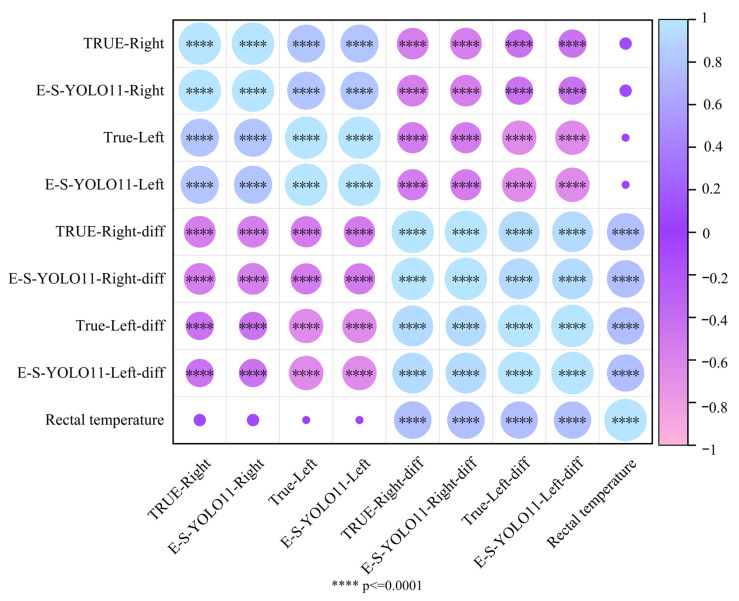
Heatmap of Pearson correlation coefficients for sheep eye temperature, rectal temperature, and their differences.

Figure 6 presents the Pearson correlation coefficients for sheep eye temperature, rectal temperature, and their differences. The results demonstrate a perfect correlation (r = 1, *p* < 0.0001) between manually annotated and E-S-YOLO11n-detected sheep eye temperature, confirming the high accuracy of the model. Additionally, regardless of the method used, the right and left eye temperatures exhibited a strong positive correlation (r = 0.8076, *p* < 0.0001), indicating a consistent trend in temperature variation between both eyes. In the comparative analysis of left and right eye temperature differences, both manual annotation and E-S-YOLO11n detection revealed a strong positive correlation between right and left eye temperature differences (r = 0.9264, *p* < 0.0001), further confirming the consistency in temperature variation between both eyes. However, the correlation between eye temperature and rectal temperature was weak (|r| < 0.0852, *p* > 0.05). Specifically, right eye temperature showed an extremely weak positive correlation with rectal temperature (r = 0.0852, *p* = 0.3924), while left eye temperature exhibited an extremely weak negative correlation (r = −0.0359, *p* = 0.7186), neither of which reached statistical significance. The Pearson correlation analysis confirms a high degree of consistency between manual annotation and E-S-YOLO11n detection for sheep eye temperature and temperature differences, further validating the model’s detection accuracy. However, the weak correlation between eye temperature and rectal temperature suggests that eye temperature variations may be independent of rectal temperature and are likely influenced by other physiological or environmental factors. Consequently, eye temperature may not serve as a direct predictor of rectal temperature, and its limitations should be carefully considered in practical applications.

Table 6 presents the t-test results for sheep eye temperature, rectal temperature, and their differences. The results indicate that rectal temperature is significantly higher than eye temperature, exceeding the right eye temperature by 7.37% and the left eye temperature by 7.69% (right eye: t = 71.61, *p* < 0.0001; left eye: t = 71.73, *p* < 0.0001). However, there was no significant difference between the eye temperatures detected by manual annotation and E-S-YOLO11n (t = 0.00, *p* = 1.0000), confirming the model’s reliability in replicating manual annotations. When comparing both eyes, the right eye temperature was slightly higher than the left, with an average difference of −0.11 °C, a statistically significant difference (t = −3.30, *p* = 0.0012 < 0.01). Additionally, a significant difference was observed between eye temperature and its corresponding temperature difference for both the right eye (t = −797.01, *p* < 0.0001) and the left eye (t = −716.55, *p* < 0.0001). Overall, the absence of a significant difference between manual annotation and E-S-YOLO11n detection highlights the model’s potential as a reliable alternative to manual annotation. However, the significant temperature difference between the right and left eyes suggests that asymmetrical cerebral blood flow and environmental factors may contribute to this variation. Moreover, the significant difference between eye and rectal temperatures indicates that while temperature is regulated by systemic physiological mechanisms, eye temperature is more susceptible to local environmental influences, highlighting the need for careful consideration when using eye temperature as a substitute for rectal temperature in physiological assessments.

## 4. Discussion

This study comprehensively evaluates the performance of E-S-YOLO11n in sheep binocular detection compared to other object detection models. The results demonstrate that E-S-YOLO11n outperforms other models in key indicators such as precision, recall, *mAP*@0.5, and *F*_1_, while simultaneously showcasing significant lightweight advantages in terms of parameters, model size, and GFLOPs. These findings validate its potential as a high-precision, high-efficiency model for sheep eye detection. E-S-YOLO11n enhances thermal infrared feature extraction for sheep eyes by integrating an EfficientNetV2-based Backbone network. EfficientNetV2, with its optimized scaling coefficients and efficient feature extraction capabilities, enables more precise acquisition and representation of thermal infrared features in the sheep eye. Additionally, the Slim-Neck architecture incorporated in the Neck network enhances feature fusion while reducing redundant computations, effectively lowering the model’s computational complexity. This design retains high detection accuracy while significantly improving inference speed, making the model more efficient and practical for real-world applications. Improvements in both the Backbone and Neck structures enable E-S-YOLO11n to outperform YOLO11n across multiple indicators, with increases in precision (2.72%), recall (2.07%), *mAP*@0.5 (1.64%), *F*_1_ (2.40%), and FPS (3.23%). These enhancements further validate the effectiveness of the model’s structural refinements.

While YOLO10n achieves a higher FPS than E-S-YOLO11n, its precision and recall suffer a noticeable decline, indicating that its speed gains come at the expense of detection accuracy. SSD, with a precision of only 69.91%, exhibits the weakest performance among all models, highlighting the limitations of its VGG16-based backbone in effectively extracting features for sheep eye detection. Moreover, YOLOx records the lowest recall, *mAP*@0.5, and *F*_1_, suggesting that its anchor-free mechanism does not offer advantages in this specific task of sheep eye detection. Although Faster R-CNN performs well in certain indicators, its large parameters, bulky model size, and slow detection speed make it less suitable for real-time applications. Its FPS of just 37.64 frame/s is well below the 322.58 frame/s achieved by E-S-YOLO11n. The Faster R-CNN struggles to meet the demands of time-critical scenarios. As a two-stage detection model, Faster R-CNN separately processes region proposal refinement and classification, improving robustness in complex environments but significantly increasing computational load and inference time.

In contrast, E-S-YOLO11n, as a one-stage detection model, strikes a balance between performance and efficiency, making it more practical for real-world applications. Additionally, E-S-YOLO11n excels in lightweight design. Compared to Centernet, it reduces the parameters and model size by 94.16% and 96.80%, respectively, while achieving a 99.71% reduction in GFLOPs compared to Faster R-CNN. This exceptional computational efficiency not only reduces hardware requirements but also enhances E-S-YOLO11n’s adaptability to edge devices, all while maintaining high precision and efficiency. These advantages make it a practical solution for large-scale farming applications. In summary, E-S-YOLO11n achieves an optimal balance between performance and efficiency in sheep eye detection. The model significantly improves detection precision and recall while reducing computational costs. This enhances its adaptability to diverse environments and compatibility with edge devices. These advancements not only support the broader adoption of thermal imaging technology in livestock farming but also provide valuable insights for future research and development in the field.

In this study, the maximum right eye temperature of sheep was 36.37 ± 0.23 °C, while the maximum left eye temperature was 36.26 ± 0.25 °C, with the right eye being 0.11 °C higher than the left eye (*p* < 0.01). Crisóstomo et al. [29] evaluated lamb growth performance based on residual feed intake (RFI) and residual intake and gain (RIG). The average left eye temperature of high-efficiency lambs was 34.40 ± 0.30 °C (RFI) and 34.60 ± 0.20 °C (RIG). The study used specialized software to annotate lamb eyes and calculated the average temperature of all pixels within the annotation box, which was lower than the maximum eye temperature. Further research indicated that high-efficiency lambs generally had lower body temperatures [30]. Corrales-Hlinka et al. [31] found that nutritionally sufficient ewes had a maximum left eye temperature of 37.10 ± 0.10 °C during shearing, whereas nutritionally deficient ewes had a maximum left eye temperature of 37.00 ± 0.10 °C. The stress triggered by shearing led to an increase in eye temperature, which explains why the left eye temperature of these ewes was 2.32% and 2.04% higher, respectively, than that observed in this study. Bakker et al. [32] measured temperature variations in different eye regions of sheep and found that the maximum temperature of the right lacrimal caruncle was 37.20 °C, while that of the left lacrimal caruncle was 36.70 °C. Compared to the maximum eye temperatures recorded in this study, these values were 2.28% and 1.21% higher, respectively. Since these values represent median eye temperatures, there is an inherent difference compared to the maximum eye temperatures recorded in this study.

Additionally, the right lacrimal caruncle temperature was higher than the left, consistent with the bilateral eye temperature difference observed in this study. The experimental environment in this study was a semi-enclosed livestock facility, where the right side of the measurement passage was enclosed by a solid wall, while the left side was open with railings. This asymmetry in environmental exposure resulted in the right eye temperature being more stable and slightly higher than the left eye temperature. Shu et al. [33] observed that in heat-stressed dairy cows, both the average and maximum temperatures of the left eye were higher than those of the right eye. In contrast, Jansson et al. [34] found no significant temperature difference between the left and right eyes in horses. These findings suggest that the influence of cerebral blood flow lateralization on bilateral eye temperature differences remains inconclusive. In addition to environmental factors, the observed asymmetry may also be associated with neurophysiological lateralization [35]. The two hemispheres of the brain can regulate thermoregulatory and vascular responses differently, potentially leading to temperature differences between the eyes [36]. Future studies could further explore this hypothesis through neurological investigations.

This study found that sheep rectal temperature was 7.37% and 7.69% higher than right and left eye temperatures, respectively (*p* < 0.0001), which is consistent with previous research. Ibáñez et al. [37] measured a rectal temperature of 38.88 ± 0.49 °C in sheep, while the temperature at the lachrymal caruncle was 36.97 ± 1.01 °C, indicating that rectal temperature was 5.17% higher than lachrymal caruncle temperature. Oliveira et al. [38] reported that under adequate hydration conditions, sheep had a rectal temperature of 39.16 ± 0.04 °C, whereas their body surface temperature was only 31.45 ± 0.17 °C, resulting in a 24.52% difference between rectal and surface temperatures. Vieira et al. [39] recorded a sheep rectal temperature of 39.09 ± 0.10 °C and a body surface temperature of 35.40 ± 0.24 °C, with a difference of 10.42% between the two. These findings collectively demonstrate that eye temperature measured using IRT is lower than rectal temperature obtained through invasive methods. Rectal temperature is a more accurate representation of core body temperature. The core body temperature is primarily maintained by metabolic heat production from internal organs and tissues, keeping it relatively stable and significantly higher than peripheral temperature. During thermoregulation, heat exchange at the skin surface is influenced by blood flow regulation, ambient temperature, and heat dissipation mechanisms, leading to greater fluctuations in peripheral temperature and making it consistently lower than core temperature [40].

This study found a weak correlation between eye temperature and rectal temperature in sheep (right eye: r = 0.0852, *p* = 0.3924; left eye: r = −0.0359, *p* = 0.7186). This result is consistent with the findings of Francesca Arfuso et al. [41], who reported a low correlation between eye temperature and rectal temperature in ewes based on Pearson correlation analysis (r = 0.19, *p* = 0.43). Similarly, Sun et al. [42] conducted a Pearson correlation analysis in sheep and observed a weak correlation between eye and rectal temperatures (r = 0.21, *p* < 0.05). However, the correlation between eye and rectal temperatures may vary under specific environmental conditions. Marques et al. [43] found a strong correlation between maximum eye temperature and rectal temperature in goats under heat stress (r = 0.956, *p* < 0.05). Likewise, Pulido-Rodríguez et al. [44] studied thermoregulation in hybrid sheep in hot environments and reported a significant positive correlation between eye and rectal temperatures (r = 0.71, *p* < 0.05).

Existing research has attempted to evaluate the feasibility of using peripheral temperature to predict core temperature in sheep. However, the correlation between eye and rectal temperatures varies depending on factors such as species, age, health status, eye region, and physiological regulatory mechanisms [45]. Notably, the lacrimal caruncle of the sheep’s eye is supplied by the infraorbital artery and is highly sensitive to vasomotor changes. It is regulated by sympathetic adrenergic fibers, making it a reliable predictor of heat stress [46]. Vasomotor responses play a crucial role in thermoregulation in most animals by modulating the diameter of peripheral blood vessels to facilitate heat exchange with the environment [47,48]. Consequently, under heat stress, eye temperature in sheep often exhibits a stronger correlation with rectal temperature. In this study, both eye and rectal temperature measurements were conducted between 5:00 and 9:00 a.m. (Beijing Time), with ambient temperatures remaining below 20 °C. The relatively low environmental temperature may have reduced heat stress in the sheep, thereby influencing the correlation between eye and rectal temperatures. This factor could explain the weak correlation observed in this study. The weak correlation between bilateral eye temperatures and rectal temperature may be attributed to several physiological and environmental factors. The eye region is a peripheral area where heat exchange is more dynamic and is heavily influenced by ambient temperature, airflow, humidity, and exposure to direct sunlight. Blood flow to the ocular region is regulated through vasodilation and vasoconstriction during thermoregulation, which introduces further variability in surface temperature. In contrast, rectal temperature represents core metabolic heat and remains relatively stable across varying conditions. This discrepancy makes eye temperature less reliable as a proxy for core body temperature, particularly in mild or semi-controlled environments where thermoregulatory responses are subtle.

The weak correlation between bilateral eye and rectal temperatures in this study suggests that eye temperature alone may not reliably serve as a proxy for rectal temperature in sheep, especially under comfortable conditions. Future research should focus on integrating multimodal data, including environmental parameters, behavioral indicators, and physiological signals, with thermal imaging to improve the accuracy of core temperature prediction. These parameters capture both environmental and physiological influences on thermoregulation. Embedding them within deep learning models could enable the development of more precise, practical, non-contact systems for real-time sheep health monitoring. Moreover, longitudinal studies involving different breeds, age groups, and management conditions are needed to validate the generalizability of eye temperature as a health indicator across diverse sheep populations.

## 5. Conclusions

The E-S-YOLO11n model, incorporating EfficientNetV2 and a Slim-Neck structure, provides a feasible solution for the rapid and automated collection of sheep bilateral eye temperature. Compared to YOLO11n, YOLO10n, YOLOx, Centernet, SSD, and Faster R-CNN, the E-S-YOLO11n model demonstrates superior performance in detecting bilateral eyes of sheep. It improves precision, recall, *mAP*@0.5, *F*_1_, and FPS while reducing parameters, model size, and GFLOPs, confirming the effectiveness of its lightweight design. The E-S-YOLO11n model achieves eye temperature measurements consistent with manual annotation. Moreover, the MAE between E-S-YOLO11n-detected eye temperature and rectal temperature aligns with that of manually annotated data, confirming the model’s potential as an alternative to manual annotation. However, the correlation between eye and rectal temperatures in sheep is weak, as eye temperature is highly susceptible to environmental influences. Consequently, eye temperature alone is unsuitable as a direct predictor of rectal temperature. The practical applications of temperature monitoring in sheep should account for environmental factors such as ambient temperature, humidity, wind speed, and wind direction. Future work should incorporate environmental covariates and develop adjustment models, to improve the predictive accuracy of rectal temperature based on eye temperature. Notably, the right eye temperature of sheep is significantly higher than the left, which may be related to brain structure and environmental influences. This asymmetry may be influenced by neurophysiological lateralization, where the brain hemispheres exhibit functional differences that impact blood flow and thermoregulation. Further investigation into the underlying neural mechanisms may provide deeper insight into sheep thermoregulation and temperature detection. Additionally, the significant difference between eye temperature and rectal temperature suggests that eye temperature is more susceptible to local environmental variations. To reduce the influence of local environmental variations on eye temperature, future studies may consider strategies such as bilateral eye temperature averaging or standardizing measurements by time of day. These approaches could improve the stability and reliability of eye temperature as a physiological indicator.

## Figures and Tables

**Table 1:** Division of the sheep thermal image dataset.

Dataset	Number of Sheep	Number of Sheep Thermal Images
Train	848	1696
Val	211	422
Test	106	212
Total	1165	2330

**Table 2:** Performance evaluation of the YOLO11 model.

Model	P/%	R/%	*mAP*@0.5/%	*F*_1_	FPS/Frame·s^−1^	Parameters/M	Size/MB	GFLOPs
YOLO11n	95.6	96.5	97.8	96.05	312.50	9.85	5.23	3.20
YOLO11s	95.3	96.6	98.9	95.95	151.52	35.91	18.30	10.74
YOLO11m	93.1	92.4	97.8	92.75	149.25	76.41	38.60	33.95
YOLO11l	82.5	89.5	92.6	85.86	119.05	96.44	48.80	43.50
YOLO11x	93.6	96.1	98.6	94.83	103.09	216.79	109.00	97.52

**Table 3:** Performance comparison of different models in sheep eye detection.

Model	P/%	R/%	*mAP*@0.5/%	*F*_1_	FPS/Frame·s^−1^	Parameters/M	Size/MB	GFLOPs
E-S-YOLO11n	98.20	98.50	99.40	98.35	322.58	7.27	3.97	1.38
YOLO11n	95.60	96.50	97.80	96.05	312.50	9.85	5.23	3.20
YOLO10n	92.80	87.70	94.90	90.18	434.78	10.28	5.50	4.16
YOLOx	71.49	58.49	77.66	64.34	129.91	34.10	34.40	13.32
Centernet	94.56	88.21	97.66	91.27	178.67	124.60	124.00	25.31
SSD	69.91	86.80	89.52	77.44	212.30	91.09	91.20	30.48
Faster R-CNN	70.42	92.93	92.62	80.12	37.64	107.90	108.00	469.66

**Table 4:** The Kolmogorov–Smirnov test for sheep eye temperature, rectal temperature, and their differences.

Parameters	Normal Parameters		Test Statistic	Asymp.Sig.
	Mean	Std. Deviation		
True-Right	36.37	0.23	0.098	0.017
E-S-YOLO11n-Right	36.37	0.23	0.098	0.017
True-Left	36.26	0.25	0.095	0.023
E-S-YOLO11n-Left	36.26	0.25	0.095	0.023
True-Right-diff	2.67	0.36	0.051	0.200
E-S-YOLO11n-Right-diff	2.67	0.36	0.051	0.200
True-Left-diff	2.78	0.39	0.052	0.200
E-S-YOLO11n-Left-diff	2.78	0.39	0.052	0.200
RT	39.05	0.30	0.053	0.200

**Table 5:** Levene’s test for sheep eye temperature, rectal temperature, and their differences.

Parameters	Levene Statistic	df1	df2	Sig.
True-Right	0.247	3	99	0.863
E-S-YOLO11n-Right	0.247	3	99	0.863
True-Left	2.621	3	99	0.055
E-S-YOLO11n-Left	2.621	3	99	0.055
True-Right-diff	0.692	3	99	0.503
E-S-YOLO11n-Right-diff	0.692	3	99	0.503
True-Left-diff	0.657	3	99	0.581
E-S-YOLO11n-Left-diff	0.657	3	99	0.581
RT	1.092	3	99	0.356

**Table 6:** The *t*-test for sheep eye temperature, rectal temperature, and their differences.

Sample		Descriptive Statistics			*t* Test	
Sample 1	Sample 2	Sample 1 Mean	Sample 2 Mean	Mean	Sample 1	Sample 2
True-Right	RT	36.37	39.05	2.68	71.61	<0.0001
E-S-YOLO11n-Right	RT	36.37	39.05	2.68	71.61	<0.0001
True-Left	RT	36.26	39.05	2.79	71.73	<0.0001
E-S-YOLO11n- Left	RT	36.26	39.05	2.79	71.73	<0.0001
True-Right-diff	RT	2.67	39.05	36.38	781.64	<0.0001
E-S-YOLO11n-Right-diff	RT	2.67	39.05	36.38	781.64	<0.0001
True-Left-diff	RT	2.78	39.05	36.27	732.97	<0.0001
E-S-YOLO11n-Left-diff	RT	2.78	39.05	36.27	732.97	<0.0001
True-Right	E-S-YOLO11n-Right	36.37	36.37	0.00	0.00	1.0000
True-Left	E-S-YOLO11n-Left	36.26	36.26	0.00	0.00	1.0000
True-Right	True-Left	36.37	36.26	−0.11	−3.30	0.0012
E-S-YOLO11n-Right	E-S-YOLO11n- Left	36.37	36.26	−0.11	−3.30	0.0012
True-Right-diff	True-Left-diff	2.67	2.78	0.11	2.08	0.0389
E-S-YOLO11n-Right-diff	E-S-YOLO11n-Left-diff	2.67	2.78	0.11	2.08	0.0389
True-Right	True-Right-diff	36.37	2.67	−33.70	−797.01	<0.0001
E-S-YOLO11n-Right	E-S-YOLO11n-Right-diff	36.37	2.67	−33.70	−797.01	<0.0001
True-Left	True-Left-diff	36.26	2.78	−33.48	−716.55	<0.0001
E-S-YOLO11n-Left	E-S-YOLO11n-Left-diff	36.26	2.78	−33.48	−716.55	<0.0001

## Data Availability

Dataset available on request from the authors.

## Abbreviations

The following abbreviations are used in this manuscript:

IRT	Infrared Thermography
Conv2D	2D Convolution
BN	Batch Normalization
DWConv	Squeeze and Excitation
SE	Linear Dichroism
True-Right	Manually Annotated Right Eye Temperature
E-S-YOLO11n-Right	Model-Detected Right Eye Temperature
True-Left	Manually Annotated Left Eye Temperature
E-S-YOLO11n-Left	Model-Detected Left Eye Temperature
True-Right-diff	Rectal Temperature Minus Manually Annotated Right Eye Temperature
E-S-YOLO11n-Right-diff	Rectal Temperature Minus Model-Detected Right Eye Temperature
True-Left-diff	Rectal Temperature Minus Manually Annotated Left Eye Temperature
E-S-YOLO11n-Left-diff	Rectal Temperature Minus Model-Detected Left Eye Temperature
RT	Rectal Temperature
K-S	Kolmogorov–Smirnov

## References

[1] Samara E.M. (2024). Profiling the dynamic variations in body and scrotal surface temperatures of goats reared under stressful conditions using infrared thermography: Analytical perspectives. J. Therm. Biol..

[2] Pacheco V.M., de Sousa R.V., Rodrigues A.V.S., Sardinha E.J.S., Martello L.S. (2020). Thermal imaging combined with predictive machine learning based model for the development of thermal stress level classifiers. Livest. Sci..

[3] George W.D., Godfrey R.W., Ketring R.C., Vinson M.C., Willard S.T. (2014). Relationship among eye and muzzle temperatures measured using digital infrared thermal imaging and vaginal and rectal temperatures in hair sheep and cattle1. J. Anim. Sci..

[4] Theusme C., Macías-Cruz U., Castañeda-Bustos V., López-Baca M.A., García-Cueto R.O., Vicente-Pérez R., Mellado M., Vargas-Villamil L., Avendaño-Reyes L. (2023). Holstein heifers in desert climate: Effect of coat color on physiological variables and prediction of rectal temperature. Trop. Anim. Health Prod..

[5] Theusme C., Avendaño-Reyes L., Macías-Cruz U., Castañeda-Bustos V., García-Cueto R., Vicente-Pérez R., Mellado M., Meza-Herrera C., Vargas-Villamil L. (2022). Prediction of rectal temperature in Holstein heifers using infrared thermography, respiration frequency, and climatic variables. Int. J. Biometeorol..

[6] Poku R.A., Agyemang-Duah E., Donkor S., Ayizanga R.A., Osei-Amponsah R., Rekaya R., Aggrey S.E. (2024). Changes in rectal temperature as a means of assessing heat tolerance and sensitivity in chickens. Trop. Anim. Health Prod..

[7] Mota-Rojas D., Titto C.G., Orihuela A., Martínez-Burnes J., Gómez-Prado J., Torres-Bernal F., Flores-Padilla K., Carvajal-de la Fuente V., Wang D. (2021). Physiological and behavioral mechanisms of thermoregulation in mammals. Animals.

[8] Giannetto C., Cerutti R.D., Scaglione M.C., Fazio F., Aragona F., Arfuso F., Zumbo A., Piccione G. (2022). Simultaneous recording of subcutaneous temperature and total locomotor activity in Bos taurus and Bos indicus raised in a subtropical region of Argentina. Trop. Anim. Health Prod..

[9] Moe A.S.T., Zin T.T., Aikawa M., Kobayashi I. Automatic Body Temperature Detection in Calves and Alarm System Using Thermographic Camera. Proceedings of the 16th International Conference on Genetic and Evolutionary Computing (ICGEC 2024).

[10] Aragona F., Rizzo M., Arfuso F., Acri G., Fazio F., Piccione G., Giannetto C. (2024). Eye temperature measured with infrared thermography to assess stress responses to road transport in horses. Animals.

[11] Giro A., Bernardi A.C.C., Junior W.B., Lemes A.P., Botta D., Romanello N., Barreto A.N., Garcia A.R. (2019). Application of microchip and infrared thermography for monitoring body temperature of beef cattle kept on pasture. J. Therm. Biol..

[12] Chen X., Ogdahl W., Hulsman Hanna L.L., Dahlen C.R., Riley D.G., Wagner S.A., Berg E.P., Sun X. (2021). Evaluation of beef cattle temperament by eye temperature using infrared thermography technology. Comput. Electron. Agric..

[13] Neto G.A.C., Machado N.A.F., Barbosa-Filho J.A.D., Marques J.I., Leite P.G., de Andrade H.A.F., de Sousa A.M., Dos Santos J.C.S., de Sousa A.C., Da Silva Sousa W. (2025). Infrared thermography as a non-invasive method to quantify the heat stress response in weaned piglets after road transport in a semi-arid region. Int. J. Biometeorol..

[14] Samara E.M., Al-Badwi M.A., Abdoun K.A., Al-Haidary A.A. (2024). Applicability of thermography as a potential non-invasive technique to assess the body-thermal status of heat-stressed and water-deprived goats (*Capra hircus*). J. Therm. Biol..

[15] Pereira A.L.V., Martello L.S., Campos J.C.D., Rodrigues A.V.S., Oliveira G.P.C.N., Sousa R.V. (2024). Predictive models for heat stress assessment in Holstein dairy heifers using infrared thermography and machine learning. Trop. Anim. Health Prod..

[16] Goursot C., Düpjan S., Puppe B., Leliveld L.M.C. (2021). Affective styles and emotional lateralization: A promising framework for animal welfare research. Appl. Anim. Behav. Sci..

[17] Wang Y., Chu M., Kang X., Liu G. (2024). A deep learning approach combining DeepLabV3+ and improved YOLOv5 to detect dairy cow mastitis. Comput. Electron. Agric..

[18] Uddin J., McNeill D.M., Lisle A.T., Phillips C.J.C. (2020). A sampling strategy for the determination of infrared temperature of relevant external body surfaces of dairy cows. Int. J. Biometeorol..

[19] Idris M., Sullivan M., Gaughan J.B., Phillips C.J.C. (2024). The Relationship between the infrared eye temperature of beef cattle and associated biological responses at high environmental temperatures. Animals.

[20] Zhang Y., Liu G., Wang J. (2025). Automated detection of sheep eye temperature using thermal images and improved YOLOv7. Comput. Electron. Agric..

[21] Wang P., Wu P., Wang C., Huang X., Wang L., Li C., Niu Q., Li H. (2025). Chicken body temperature monitoring method in complex environment based on multi-source image fusion and deep learning. Comput. Electron. Agric..

[22] Zhang B., Xiao D., Liu J., Huang S., Huang Y., Lin T. (2024). Pig eye area temperature extraction algorithm based on registered images. Comput. Electron. Agric..

[23] Jeyakumar S., Kumaresan A., Kataktalware M.A., Manimaran A., Ramesha K.P. (2022). Frontier Technologies in Bovine Reproduction: Infrared Thermal Imaging and Its Application in Animal Reproduction.

[24] Stumpf M.T., McManus C.M., Daltro D.S., Alfonzo E.P.M., Dalcin V., Kolling G.J., Vieira R.A., Louvandini H., Fischer V., da Silva M.V.G.B. (2020). Different methods of assessing udder temperature through thermography and their relation with rectal temperature. Trop. Anim. Health Prod..

[25] Easterwood L., Cohen N.D. (2023). Agreement of temperatures measured using a non-contact infrared thermometer with a rectal digital thermometer in horses. J. Equine Vet. Sci..

[26] Hoffman A.A., Long N.S., Carroll J.A., Burdick Sanchez N.C., Broadway P.R., Richeson J.T., Jackson T.C., Hales K.E. (2023). Infrared thermography as an alternative technique for measuring body temperature in cattle. Appl. Anim. Sci..

[27] Tan M., Le Q. EfficientNetV2: Smaller models and faster training. Proceedings of the 38th International Conference on Machine Learning.

[28] Li H., Li J., Wei H., Liu Z., Zhan Z., Ren Q. (2024). Slim-neck by GSConv: A lightweight-design for real-time detector architectures. J. Real Time Image Process..

[29] Crisóstomo C., Bernardi R.F., Gurgeira D.N., Silveira R.M.F., Vicentini R.R., Márquez S.P., Abdalla A.L., Paro De Paz C.C., Ferreira J., Dias Da Costa R.L. (2025). Relationship between body temperature measured by infrared thermography and performance, feed efficiency and enteric gas emission of hair lambs. J. Therm. Biol..

[30] Schaefer A.L., Iheshiulor O., von Gaza H., Charagu P., Simpson G., Huisman A. (2023). Thermal Profiles: Novel phenotypic measurements of animal growth and metabolic efficiency. J. Therm. Biol..

[31] Corrales-Hlinka F., Freitas-de-Melo A., Ungerfeld R., Pérez-Clariget R. (2023). Thermoregulatory, metabolic and stress responses to spring shearing of aged ewes born to undernourished mothers. J. Therm. Biol..

[32] Bakker M.L., Milano G.D., Fernández J., Alvarado P.I., Nadin L.B. (2024). Lack of agreement among analysers of infrared thermal images in the temperature of eye regions in sheep. J. Therm. Biol..

[33] Shu H., Li Y., Fang T., Xing M., Sun F., Chen X., Bindelle J., Wang W., Guo L. (2022). Evaluation of the best region for measuring eye temperature in dairy cows exposed to heat stress. Front. Vet. Sci..

[34] Jansson A., Lindgren G., Velie B.D., Solé M. (2021). An investigation into factors influencing basal eye temperature in the domestic horse (*Equus caballus*) when measured using infrared thermography in field conditions. Physiol. Behav..

[35] Goma A.A., Uddin J., Kieson E. (2023). Lateralised behavioural responses in livestock to environmental stressors: Implications for using infrared thermography to assess welfare conditions. Animals.

[36] Zhang C.Y., Kindell M., Meagher R.K. (2025). Are laterality effects present in novel objectresponses of calves?. Appl. Anim. Behav. Sci..

[37] Ibáñez C., Moreno-Manrique M., Villagrá A., Bueso-Ródenas J., Mínguez C. (2024). Evaluation of Non-Contact Device to Measure Body Temperature in Sheep. Animals.

[38] Oliveira B.Y.S., Moura C.M.S., Araújo G.G.L., Turco S.H.N., Voltolini T.V., Furtado D.A., Medeiros A.N., Gois G.C., Campos F.S. (2024). Thermoregulatory responses and ingestive behavior of sheep subjected to water restriction and high- and low-energy diets in a semi-arid environment. J. Therm. Biol..

[39] Vieira D.S., Oliveira J.S., Santos E.M., dos Santos B.R.C., Pinto L.F.B., Zanine A.M., Coelho D.F.O., Sobral G.C., Leite G.M., Soares R.L. (2022). Microbiological composition of diets of cactus pear-based with increasing levels of buffel grass hay and relationship to nutritional disorders in sheep. Animals.

[40] Kearton T.R., Doughty A.K., Morton C.L., Hinch G.N., Godwin I.R., Cowley F.C. (2020). Core and peripheral site measurement of body temperature in short wool sheep. J. Therm. Biol..

[41] Arfuso F., Acri G., Piccione G., Sansotta C., Fazio F., Giudice E., Giannetto C. (2022). Eye surface infrared thermography usefulness as a noninvasive method of measuring stress response in sheep during shearing: Correlations with serum cortisol and rectal temperature values. Physiol. Behav..

[42] Sun L., Liu G., Jiang X. (2024). Relationships of infrared thermography temperature with core temperature in goat. Trop. Anim. Health Prod..

[43] Marques J., Leite P., Neto J., Furtado D., Lopes F. (2021). Estimation of rectal temperature of goats based on surface temperature. Eng. Agrícola.

[44] Pulido-Rodríguez L.F., Titto C.G., Bruni G.D.A., Froge G.A., Fuloni M.F., Payan-Carrera R., Henrique F.L., Geraldo A.C.A.P., Pereira A.M.F. (2021). Effect of solar radiation on thermoregulatory responses of Santa Inês sheep and their crosses with wool and hair Dorper sheep. Small Rumin. Res..

[45] Knight M.I., Linden N.P., Butler K.L., Rice M., Ponnampalam E.N., Behrendt R., Jongman E.C. (2023). The effect of shade on sheep grazing pasture during summer conditions. J. Vet. Behav..

[46] Ghezzi M.D., Napolitano F., Casas-Alvarado A., Hernández-Ávalos I., Domínguez-Oliva A., Olmos-Hernández A., Pereira A.M.F. (2024). Utilization of Infrared Thermography in Assessing Thermal Responses of Farm Animals under Heat Stress. Animals.

[47] Yamin D., Beena V., Ramnath V., Zarina A., Harikumar S., Venkatachalapathy R.T., Gleeja V.L. (2022). Impact of thermal stress on physiological, behavioural and biochemical parameters in native and crossbred goats. Small Rumin. Res..

[48] Peng D., Chen S., Li G., Chen J., Wang J., Gu X. (2019). Infrared thermography measured body surface temperature and its relationship with rectal temperature in dairy cows under different temperature-humidity indexes. Int. J. Biometeorol..

